# Spatial–Temporal Characteristics of Illegal Land Use and Its Driving Factors in China from 2004 to 2017

**DOI:** 10.3390/ijerph18031336

**Published:** 2021-02-02

**Authors:** Hongwei Zhang, Zhanqi Wang, Bin Yang, Ji Chai, Chao Wei

**Affiliations:** 1 School of Public Administration, China University of Geosciences, Wuhan 430074, China; zhangfocus@cug.edu.cn (H.Z.); cugyangbin2011@163.com (B.Y.); chaiji_cug@163.com (J.C.); 2 School of Politics, Law and Public Administration, Hubei University, Wuhan 430000, China; weichao@cug.edu.cn

**Keywords:** spatial–temporal characteristics, illegal land use, driving factors, China

## Abstract

The scientific analysis of spatial-temporal differentiation characteristics and driving factors of illegal land use is of great significance for the formulation and optimization of policies to control the emergence of illegal land use. This paper establishes two variable systems of illegal land use and its driving factors, defined the multidimensional characteristic variables of illegal land use and analyzes the relationships among them by the Pearson’s correlation coefficient; In addition, the spatial–temporal characteristics of each variable of illegal land use from 2004 to 2017 are described by the spatial autocorrelation analysis; Finally, based on the geographical detectors, the influence direction and degree of the factors of economic structure, social structure and land market behavior on the characteristics of different illegal land use are studied. The results show that the spatial agglomeration of different characteristics of illegal land use had been weakening from 2004 to 2017, but the rate of weakening was different, and L-L agglomeration changed between Xinjiang and other central-western provinces, H-H agglomeration changed in the coastal regions of the central-eastern of China, the level and ability of the central government and local governments to govern illegal land use is constantly improving on the whole; the compositional factors of economic development structure, social development structure, and land market behavior of driving factors had different influence in the degree, the location or the direction of different characteristics of illegal land use. According to the spatial–temporal characteristics and the differences of driving factors, managers can formulate differentiated illegal land use control policies, which will help to control the occurrence of illegal land use and help the settlement of illegal land use cases, and ultimately achieve sustainable development.

## 1. Introduction

The contradiction between the supply of land for human production and living activities and the demand for land has become increasingly prominent with the rapid development of the global economy and society, which has led to cases of illegal land use occurring all over the world, and this phenomenon is particularly serious in developing countries with large populations and rapid economic-social development [1,2,3]. The analysis of the spatial–temporal characteristics of illegal land use and its driving factors can guide the formulation of effective policies and measures to prevent or solve illegal land use cases, and it is of great significance to promote the legal utilization of land resources and the sustainable development of mankind.

In China, the basic system of society decides that the right to allocate the land that carries main social-economic activities is in the hands of the government or village unity [4,5], and China takes advantage of this unique system to carry out strict farmland protection system and limited supply of construction land to ensure food security and the realization of sustainable development goals [6]. Nonetheless, as the total amount of social-economic activities has become larger and larger and the asset attributes of land continue to emerge, some citizens and even local governments have chosen to break through the strict economical and intensive land-use system for greater economic benefits [7,8]. Therefore, illegal land use is also common in China, and illegal land use in China refers to non-agricultural construction without approval, illegal landfilling for the implementation of non-agricultural construction, and historical illegal land use for reconstruction and expansion of legal land and other land use. In order to effectively curb illegal land use, Chinese governments had constantly strengthened institutional construction, such as constantly improving the system of economical and intensive land use, establishing land supervision institutions [9,10].

A large number of scholars had carried out researches on the spatial–temporal characteristics of illegal land use and its driving factors, which were mainly manifested in the following characteristics: First, they discussed the relationship between a single important social or economic activity and the occurrence of illegal land use cases, and these economic-social factors include the marketization reform of land supply [11,12,13], construction of land supervision system [14], the rapid growth of economy [15,16,17]; land tenure, poverty, and forest use rights [18], its impact on the environment [19] and land use planning [20,21]. The second is to focus on the characterization of illegal land use, which includes the characteristics of the encroachment of illegal land on farmland [22,23], the spatial distribution characteristics of illegal land use [24], spatial distribution characteristics of illegal land around specific geographic objects [25]. The third is to analyze the internal mechanism of illegal land use cases based on game theory [26,27]. The fourth is to identify the location of illegal land use cases on the micro scale based on geographic information systems, remote sensing images, or land use survey data [28,29,30,31]. Fifthly, some scholars had conducted research on the effects of policies and systems formulated by management agencies at all levels for illegal land use and analyzed the directions for improvement or optimization [32,33,34].

On the whole, the research on illegal land use is rich and diverse, but there is also some content that needs further research: As China has a huge economic aggregate and large population, it has always attached great importance to illegal land use cases and a series of policies and measures for illegal land use had been carried out, but how effective are these policies? It needs to be tested by analyzing the spatial–temporal characteristics of illegal land use; meanwhile, the policies and measures for the control of illegal land use need to be further optimized; In the process of characterizing the characteristics of illegal land use, single variables such as the area of illegal land and the number of illegal land use cases are often used, but there is a lack of thinking about the multidimensional characteristics of illegal land use cases, and scholars pay more attention to the impact of a single element or overall characteristic of the economy and society on the occurrence of illegal land use cases and lack a systematic study on the effect of the internal structure of economic-social factors on the occurrence of illegal land use cases.

## 2. Assumptions and Analysis Framework

Based on the analysis of the research status, this paper proposed the following assumptions. Assumption 1: The characteristics of illegal land use cannot only be represented by a single variable such as the area of land involved in illegal land use, which may involve the area of cultivated land occupied by illegal land use cases, the resolution of illegal land cases, and the land area involved in illegal land use cases. Moreover, these variables represent different characteristics of illegal land use, and there may not be a strong correlation. Assumption 2: Compared with the overall situation of economic-social development or the basic characteristics of the land market on the impact of illegal land use phenomenon, this paper believes that different factors involved in the structure of economic development, the structure of social development, and the structure of the land market behaviors, respectively that are quite different in the degree or direction of the impact on the phenomenon of illegal land use.

**Figure 1:**
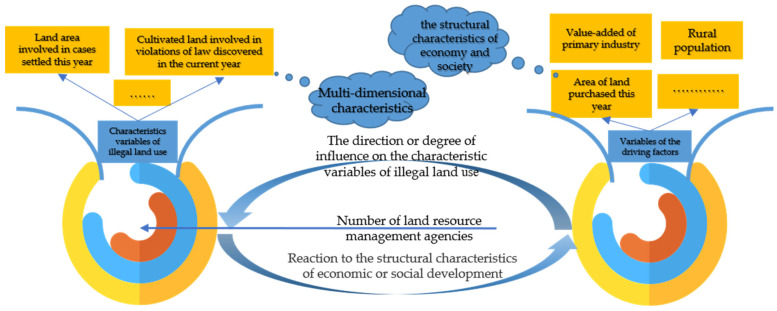
Analysis framework of spatial–temporal characteristics of illegal land use and its driving factors in China.

According to the above basic assumptions, this paper would establish a variable system describing the characteristics of illegal land use, introduced the number of land resources management agencies as an independent variable at the same time, selected variables that can fully characterize the phenomenon of illegal land use by the Pearson’s correlation coefficient, analyzed the relationship between the number of land resource management agencies and the characteristics of illegal land use, and finally described the spatial–temporal characteristics changes of different characteristics of illegal land use by spatial autocorrelation analysis. Second, this paper established a variable system of driving factors that reflected the economic development structure, social development structure and land market behavior structure, and analyzed the core driving factors that influenced the changes of different characteristics of illegal land use based on geographical detectors (Figure 1).

## 3. Material and Methods

### 3.1. Data Description

China has 34 provincial-level administrative regions (including 23 provinces, 5 autonomous regions, four municipalities directly under the central government and 2 special administrative regions). We examined 31 provincial administrative regions in China as the basic units to evaluate the spatial–temporal distribution of illegal land use from 2004 to 2017. Out of 31 provinces and autonomous regions, we excluded “Taiwan”, “Hong Kong”, and “Macao” due to the lack of the reliability and stability of the data in these regions. In addition, the data of illegal land use before 2012 was from the China Land and Resources Almanac (CLRA), but the data of illegal land use after 2012 was from China Land and Resources Statistical Yearbook (CLRSY). On the other hand, the data of economic-social development come from the China Statistical Yearbook (CSY), China Regional Statistical Yearbook (CRSY), China Real Estate Statistics Yearbook (CRESY) and statistical yearbook of different provinces (SYDP), and we unified the statistical scales and units of different data sources. However, data are not available for all provinces; we attempted the linear interpolation to fill in the missing data and provide further discussions on the variables used for the statistical analysis in the following sections.

### 3.2. Methodology

#### 3.2.1. Variable System of Illegal Land Use and Its Driving Factors

(1) Variable system of illegal land use

The multidimensional analysis of the spatial–temporal characteristics of illegal land use is helpful to scientifically describe the real effect of the policies and measures for controlling illegal land use. Based on the assumptions and analysis framework, this paper constructed the comprehensive variable system including nine variables; the number of land resources management agencies (NumLRMA) was selected as the independent variable to verify the relationship between NumLRMA and the characteristics of illegal land use at the same time. The definition and sources of specific variables are shown in Table 1.

(2) Variable system of driving factors

Previous studies have shown that the occurrence of illegal land use is closely related to economic and social level or land marketization in China [35], but which economic activity has the greatest impact on economic activities? Which social activities have had the greatest impact? Which land market behavior disturbs it the most? Therefore, this paper selected driving variables to construct a driving factor system from the aspects of the composition of economic structure, the composition of the social structure, and the structural characteristics of land market behavior. The variables of the driving factors are shown in Table 2.

#### 3.2.2. Pearson Correlation Coefficient

As mentioned above, there are many variables that represent the characteristics of illegal land use, and there may be a strong correlation among various variables, but the strength of the relationship in different variables needs quantitative verification. Based on the variable system of illegal land use, Pearson’s correlation coefficient was introduced to calculate the possible correlation of each variable, and finally, the most representative variables were selected to depict the characteristics of illegal land use. Pearson’s correlation coefficient reflects the trend of the change of two linear data groups by dividing the covariance of two variables by the standard deviation of two variables [36]. The specific formula is as follows:

(1) $$r=\frac{\sum_{i=1}^{n}x_{i}y_{i}-\frac{\sum_{i=1}^{n}x_{i}\sum_{i=1}^{n}y_{i}}{n}}{\sqrt{\sum_{i=1}^{n}x_{i}^{2}-\frac{(\sum_{i=1}^{n}x_{i}{)}^{2}}{n}}\sqrt{\sum_{i=1}^{n}y_{i}^{2}-\frac{{\left(\sum_{i=1}^{n}y_{i}\right)}^{2}}{n}}}$$

#### 3.2.3. Spatial Autocorrelation Analysis

The method was selected to characterize the spatial–temporal characteristics of illegal land use was spatial autocorrelation analysis; spatial autocorrelation refers to the potential interdependencies between the data of some variables within the same distribution area, spatial autocorrelation statistics are a fundamental property used to measure geographic data: the degree of interdependence between data at one location and data at other location. Global Moran’s I and local Moran’s I are two spatial autocorrelation representations that are used to characterize the spatial autocorrelation, and this paper would select these to measure the spatial autocorrelation characteristics of illegal land use over the years. The specific calculation formulas are as follows:

(1) Global Moran’s I

Global Moran’s I is an indicator used to evaluate whether the expressed mode is an aggregation mode, a discrete mode, or a random mode in the entire area, and global Moran’s I can be regarded as the correlation coefficient between the observed value and its spatial lag [37]. The specific formula is as follows:

(2) $$I=\frac{n}{S_{0}}\frac{\sum_{i=1}^{n}\sum_{j=1}^{n}w_{i,j}z_{i}z_{j}}{\sum_{i=1}^{n}z_{i}^{2}}$$

In the results, the value of Moran’s I is generally between −1 and 1. If the value of global Moran’s I is positive, the positive spatial correlation of this element is strong (in particular, a high value is adjacent to a high value, and a low value is adjacent to a low value), but if the value of Moran’s I is negative, the element has a strong discrete trend, and the correlation is not very pronounced (in particular, a high value is adjacent to a low value, and a low value is adjacent to a high value). If Moran’s I is close to 0, it means that the attributes are randomly distributed (or there is no spatial autocorrelation).

(2) Local Moran’s I

If global Moran’s I is significant, we can assume that there is a spatial correlation in this region, but we still do not know exactly where there is spatial aggregation. Local spatial autocorrelation analysis can make up for the deficiency of global autocorrelation analysis, and local Moran’s I is an indicator to test the local spatial autocorrelation, LISA (Local indicators of spatial association) cluster map is the basic representation of local Moran’s I, and the cluster/outlier type (CO type) field distinguishes between a statistically significant cluster of high values (H-H), cluster of low values (L-L), an outlier in which a high value is surrounded primarily by low values (H-L), and an outlier in which a low value is surrounded primarily by high values (L-H). The specific calculation formula is as follows:

(3) $$I_{i}=\frac{x_{i}-\bar{X}}{S_{i}^{2}}\overset{n}{\underset{j=1,j\neq i}{\sum }}w_{i,j}\left(x_{j}-\bar{X}\right)$$

A positive $I_{i}$ indicates that it is high and surrounded by high values, or it is low and surrounded by low values; a negative $I_{i}$ indicates that it is low but surrounded by high values, or it is high and surrounded by low values.

#### 3.2.4. Geographical Detectors

Factor detection: This paper was to detect to what extent that the driving factor X explains the spatial differentiation of the characteristic variables Y of illegal land use, and the conclusion is measured by *q* value [38]. The expression is:

(4) $$q_{D,\;U}=1-\frac{1}{nu_{U}^{2}}\overset{m}{\underset{i=1}{\sum }}n_{D,\;i}\sigma_{U_{D,i}}^{2}$$

where $n_{D,\;i}$ is the number of samples in the secondary region i; *n* is the number of samples in the whole area; $u_{U}^{2}$ represents the variance of the characteristic variables of illegal land use and $\sigma_{U_{D,\;i}}^{2}\;$ represents the variance of the secondary regions i. Formally, $\sigma_{U_{D,\;i}}^{2}\neq 0$, and the range of $q_{D,\;U}$ is (0, 1). On the other hand, the larger the $q_{D,\;U}$ is, the more obvious the spatial differentiation of Y is. If the stratification is generated by the independent variable X, the larger the *q* value is, the stronger the explanatory power of the independent variable X to the attribute Y is, and conversely, the weaker it is.

Interaction detection: Identify the interaction between different driving factors X_s_, that is, assess whether the factors X_1_ and X_2_ work together to increase or decrease the explanatory power of the dependent variable Y, or the impact of these factors on Y is independent of each other. The evaluation method is to calculate the *q* values of two factors X_1_ and X_2_ to Y: *q*(X_1_) and *q*(X_2_) first, and calculate their interaction *q* value: *q*(X_1_ ∩ X_2_), and compare *q*(X_1_), *q*(X_2_) and *q*(X_1_ ∩ X_2_) [38]. The relationship between the two factors can be divided into the following categories (Table 3):

Ecological detector: Different from interactive detection, the ecological detector is used to compare whether the effects of the two driving factors X_1_ and X_2_ on the spatial distribution of the characteristic variable Y are significantly different; this paper thinks that it can effectively analyze the impact of various driving factors of illegal land use on the spatial distribution of the characteristics of illegal land use [38]. The specific formula is as follows:

(5) $$F=\frac{N_{X1}\left(N_{x2}-1\right)SSW_{X1}}{N_{X2}\left(N_{x1}-1\right)SSW_{X2}}$$

where: $N_{X1}$ and $N_{x2},$ respectively represent the sample sizes of two factors X_1_ and X_2_; $SSW_{X1}$ and $SSW_{X2},$ respectively represent the sum of the intra-layer variances of the layers formed by X_1_ and X_2_. Where null hypothesis $H_{0}$: $SSW_{X1}$=$SSW_{X2}$. If $H_{0}$ is rejected at the significance level of α, it indicates that the two factors X_1_ and X_2_ have a significantly different effect on the spatial distribution of attribute Y.

## 4. Results

### 4.1. Result of the Selection of Variables for Characterizing Illegal Land Use

On the basis of the illegal land use variable system constructed in Table 1, the method of Pearson’s correlation coefficient and the relevant data from 2004 to 2017, the correlation coefficient between the various variables were measured, and the results were as follows (Table 4):

On the whole, each characteristic variable of illegal land has a certain significant correlation; the results showed that NumLRMA of each evaluation unit did not have a high correlation with the occurrence of illegal land use. Second, CasesULY, VLDCY and CLICULY had a relatively high degree of independence with the low value of the Pearson’s correlation coefficient. Third, CasesSTY had a high correlation with other variables, which could be used as representative variables to express the characteristics of a series of related variables. Therefore, the variables of CasesULY, VLDCY, CLICULY and CasesSTY were selected to describe the characteristics of illegal land use.

### 4.2. Spatial-Temporal Characteristics of the Distribution of Illegal Land Use

According to the Pearson’s correlation coefficient matrix, four variables that included CasesULY, VLDCY, CasesSTY and CLICULY were selected to characterize the basic characterization of illegal land use, and then carried out spatial autocorrelation analysis, calculated global Moran’s I and drew LISA cluster maps of local Moran’s I about the four variables and the independent variable of NumLRMA.

#### 4.2.1. The Results of Global Moran’s I Measurement

Judging from the results of CasesULY’s global Moran’s I and critical value (z-score), it showed a certain spatial autocorrelation before 2009 that indicated that CasesULY showed the characteristics of spatial aggregation in China before 2009; judging from the results of VLDCY’s global Moran’s I and critical value (z-score), VLDCY showed a random distribution only after 2012; from the results of CasesSTY’s global Moran’s I and critical value (z-score), CasesSTY showed the characteristics of clustering distribution from 2004 to 2007; however, CLICULY showed the characteristics of clustered distribution before 2011. At the same time, global Moran’s I of NumLRMA was also calculated, and there was only a weak spatial correlation in 2004 and 2005 (Table 5).

#### 4.2.2. The Results of Local Moran’s I Measurement

**Figure 2:**
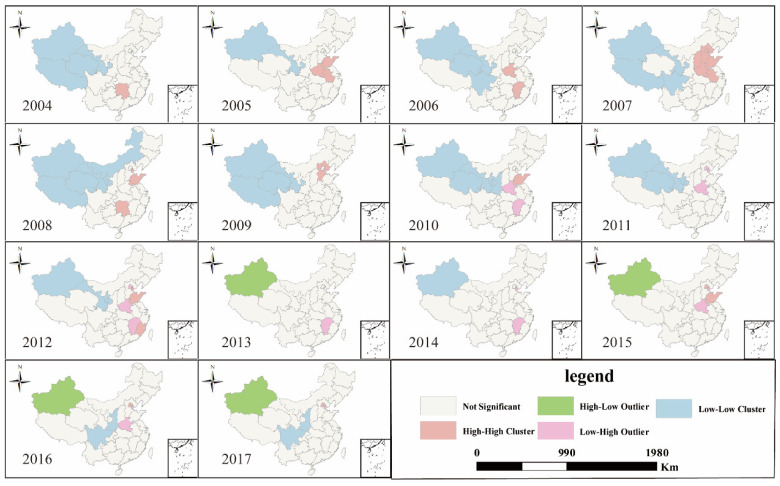
LISA (Local indicators of spatial association) cluster map of CasesULY in China from 2004 to 2017.

The agglomeration of CasesULY presented the following characteristics: it showed obvious L-L aggregation characteristics in northwest China from 2004 to 2012, and there were fewer L-L aggregation units after 2013. However, the H-H units were not obvious from 2004 to 2017; it was only in the first few years that there was a certain spatial agglomeration, and the number of H-H units reached its peak in 2007, which mainly distributed in the Beijing–Tianjin–Hebei Zone. At the same time, the L-L agglomeration units had a tendency to shift to the middle in the process of shrinking, while the H-H agglomeration units gradually disappeared, and low-high agglomeration units appeared (Figure 2).

**Figure 3:**
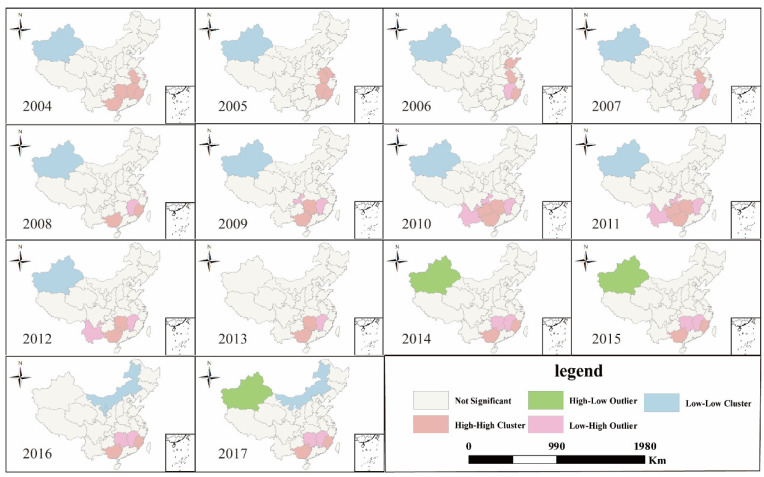
LISA (Local indicators of spatial association) cluster map of VLDCY in China from 2004 to 2017.

Judging from the LISA cluster map of VLDCY, the spatial clustering situation from 2004 to 2017 was not obvious. H-H agglomeration units were mainly concentrated in the south-west provinces such as Hunan and Guangxi province, and the number of H-H agglomeration units dropped from 5 in 2004 to 2 in 2017. L-L agglomeration units mainly changed between the two provinces of Xinjiang and Neimenggu, and there was no significant change in the number of L-L aggregation units. At the same time, the situation of high-low clustering appeared after 2014 (Figure 3).

**Figure 4:**
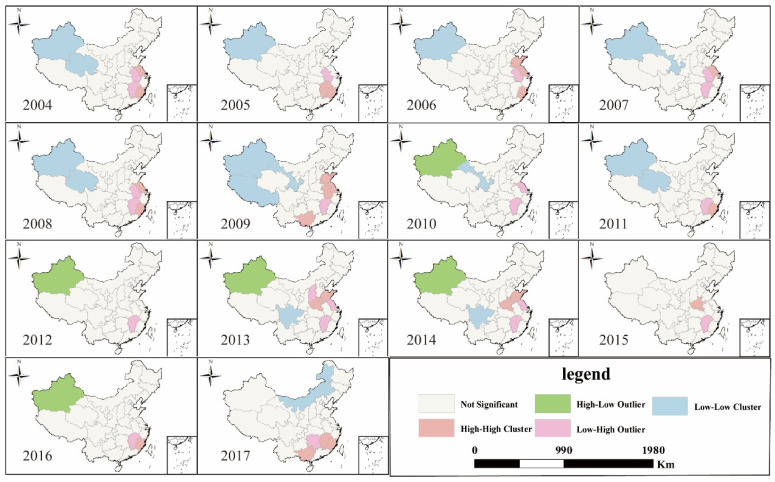
LISA (Local indicators of spatial association) cluster map of CasesSTY in China from 2004 to 2017.

The spatial agglomeration situation of CasesSTY was similar to that of VLDCY, and its spatial agglomeration situation was not obvious from 2004 to 2017. Compared with VLDCY, L-L agglomeration units were more widely distributed, and the number of units showed a trend of increasing first and then decreasing, but they were mainly concentrated in the western of China. H-H agglomeration units were mainly concentrated in eastern coastal provinces such as Shanghai, Shandong and Fujian, and the distribution area had been changing, the number of units changed little overall (Figure 4).

**Figure 5:**
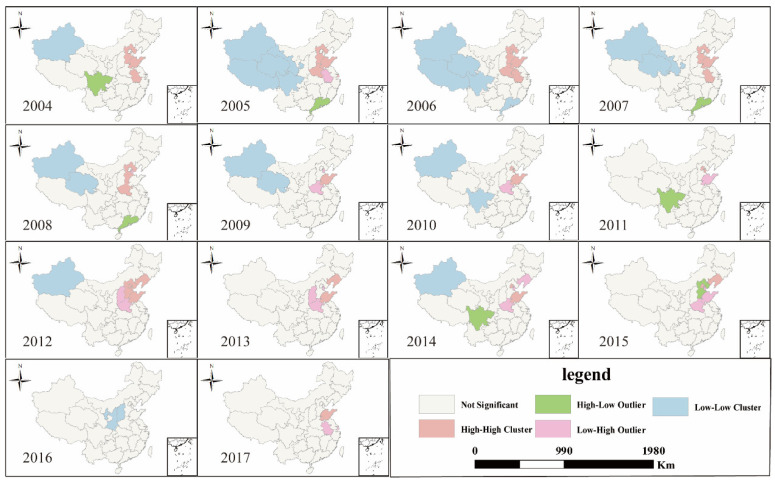
LISA (Local indicators of spatial association) cluster map of CLICULY in China from 2004 to 2017.

Compared with CasesULY, VLDCY and CasesSTY, the spatial agglomeration of CLICULY changed significantly from 2004 to 2017. The L-L agglomeration units were more widely distributed before 2010, mainly concentrated in the western region of China, but the number of L-L agglomeration units quickly became fewer after 2010. H-H agglomeration units were mainly concentrated in Shandong, Liaoning and other central-eastern coastal provinces, and their distribution units were also becoming smaller from 2004 to 2017 (Figure 5).

**Figure 6:**
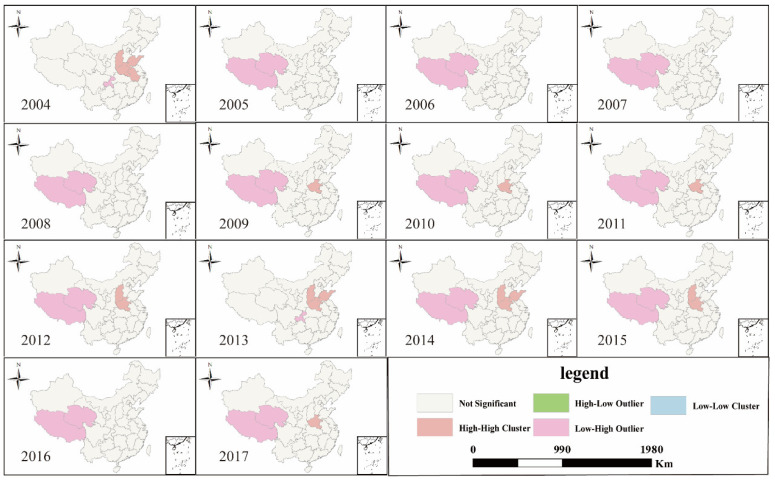
LISA (Local indicators of spatial association) cluster map of NumLRMA in China from 2004 to 2017.

At the same time, the LISA cluster map of NumLRMA was drawn, the results showed that China’s NumLRMA did not have L-L aggregation from 2004 to 2017, but there were more low-high aggregation units. In addition, H-H agglomeration units mainly fluctuated among provinces such as Henan, Shandong and Hebei, and there were few evaluation units for H-H agglomeration. Overall, it showed relatively stable characteristics and was quite different from the spatial agglomeration characteristics of CasesULY, VLDCY, CasesSTY and CLICULY (Figure 6).

### 4.3. Geographical Detection Results of Driving Factors on Illegal Land Use

#### 4.3.1. Factor Detection of Driving Factors

From the detection results of the driving factors of CasesULY, the explanatory power of Pnsch and Rp on the occurrence of CasesULY reached 0.8107 and 0.6965, respectively, but VaSin and VaTin had less influence on the occurrence of CasesULY. VLDCY was mainly affected by VaTin, Up, TNreden, ALpurchTY, LTpTY and Tarede, with explanatory power reaching 0.8797, 0.8845, 0.8768, 0.8959, 0.9139 and 0.8888, respectively. The factors that affected CasesSTY and VLDCY had a high consistency, VaTin, Up, TNreden, ALpurchTY, LTpTY and Tarede also had a greater impact on CasesSTY. However, only VaSin, VaTin, and Up had stronger explanatory power for CLICULY, reaching 0.6229, 0.6302 and 0.6132, respectively (Table 6).

#### 4.3.2. Interaction Detection of Driving Factors

From the results of interaction detection of driving factors on the variable of CasesULY of illegal land use, Ur and Up, Ur and Rp, Ur and VaTin, Ur and Upd, Ur and ALpurchTY, Upd and Up, Upd and Pnsch, etc. had a strong interaction on CasesULY. On the whole, different driving factors had a strong synergistic effect on CasesULY, but Ur and Rp had the strongest interaction among all types, which showed that social factors were the main driving factors for the occurrence of CasesULY (Table 7).

Compared with the interaction detection results of CasesULY, driving factors had a greater interaction effect on VLDCY, and the majority of the interaction influence remained above 0.8800. Among them, the values of the interaction on VaSin and VaPin, Upd and Up, Ur and Up, Ur and ALpurchTY, Ur and LTpTY reached 0.9953, 0.9948, 0.9966, 0.9919, 0.9953, respectively, but Ur and Up had the strongest interaction, which showed that social factors were the main driving factors for the occurrence of VLDCY (Table 8).

The interaction of various factors on CasesSTY was weaker than that of VLDCY but was still at a high-level as a whole. Among them, the factors with strong interaction were VaSin and VaPin, Upd and Up, Ur and Up, Ur and ALpurchTY, which was highly consistent with the interaction results of VLDCY, and its interaction forces reached 0.9898, 0.9954, 0.9897, 0.9888, respectively. However, unlike VLDCY, Upd and Up have the strongest interaction, which showed that social factors also were the main driving factors for the occurrence of CasesSTY (Table 9).

The interaction of different factors on CLICULY was weaker than that on other characteristic variables. Among them, only Upd and Up, Ur and Rp had stronger interaction, with strengths of 0.9938 and 0.9965, respectively, and Ur and Up had the strongest interaction, which showed that social factors also were the main driving factors for the occurrence of CLICULY (Table 10).

Ur and Rp, Ur and Up, Upd and Up, Ur and Up had the strongest interaction for CasesULY, VLDCY, CasesSTY, CLICULY severally, and the value reached 0.9972, 0.9966, 0.9954, 0.9719, respectively, which showed that social factors also were the main driving factors for the occurrence of illegal land use, but different characteristics of illegal land use were affected by different social structural factors.

#### 4.3.3. Results of Ecological Detector of Driving Factors

From the results of ecological exploration, the influence of Rp and Pnsch on the spatial distribution of CasesULY was significantly different from other driving factors, and two factors were mainly social factors (Table 11).

Analyzing the ecological detection results of the spatial distribution of VLDCY, the effects of VaTin, TNreden, ALpurchTY, LTpTY and Tarede on the spatial distribution of VLDCY were significantly different from other driving factors. Among them, the three driving factors of VaTin, ALpurchTY and LTpTY were more different than other factors; it indicated that the factors affecting the spatial distribution of VLDCY included economic structure factors and land market behavior factors (Table 12).

Similar to the ecological detection results of the spatial distribution of VLDCY, the influence of VaTin, TNreden, ALpurchTY, LTpTY and Tarede on the spatial distribution of CasesSTY was significantly different from other driving factors. Among them, the three driving factors of VaTin, ALpurchTY and LTpTY also were more different than other factors, which also included in economic structure factors and land market behavior factors (Table 13).

The ecological detection results of the spatial distribution of CLICULY showed that the influence of VaSin, VaTin and ALpurchTY on the spatial distribution of CLICULY was significantly different from other driving factors, which was also included in economic structure factors and land market behavior factors (Table 14).

The ecological detector discloses relative importance between the factors. On the whole, social factors have a greater impact on the occurrence of CasesULY, while economic structure factors and land market behavior factors have a greater impact on VLDCY, CasesSTY, and CLICULY.

## 5. Discussion

### 5.1. Can a Single Variable Effectively Characterize Illegal Land Use?

Based on the analysis framework and basic assumptions in Chapter 2, by constructing a characteristic variable system of illegal land use, this paper analyzed the Pearson’s correlation coefficient of panel data in each province from 2004 to 2017. The results showed that some variables were highly independent, but there were also many variables that were highly correlated, which showed that a single variable such as the number of illegal land use cases was simply chosen to characterize the occurrence of illegal land use was obviously not scientific enough. On the other hand, it is not conducive to analyzing the characteristics of illegal land use cases and the effect of a series of policies and measures for illegal land use and is also not conducive to formulate policies to promote the reduction and resolution of illegal land use cases. However, the current research is still focused on characterizing illegal land use based on a single variable; for example, Zhigang Chen and Mingchao Jia systematically studied whether the continuous development of the land market inhibited the occurrence of illegal land use cases, and only the area of illegal land use was selected to characterize illegal land use among them, the data either came from the government statistics department or obtained through remote sensing interpretation [12,30].

In this paper, four variables that included CasesULY, VLDCY, CLICULY and CasesSTY were selected as the basic representations of illegal land use measured by the Pearson’s correlation coefficient. CasesULY represents the characteristics of illegal land use cases that are more difficult to solve. CLICULY represents the area of cultivated land involved in illegal land use cases that is difficult to solve, which is the biggest threat to food security. VLDCY represents the current occurrence of illegal land use cases that can or cannot be resolved. CasesSTY represents the number of illegal land use cases that have been resolved this year. The selection of the variables can fully reflect the characteristics of illegal land use.

### 5.2. How NumLRMA Affects the Spatial–Temporal Distribution of Illegal Land Use

Whether it is Pearson’s correlation coefficient Analysis or spatial Autocorrelation Analysis, this paper introduced the variable of NumLRMA. The results showed that NumLRMA had no significant correlation with illegal land features. On the other hand, NumLRMA had almost no spatial autocorrelation features, which indicated that NumLRMA did not affect the occurrence or settlement of illegal land use. Therefore, it was obvious that the implementation strength and management level of various land resources management agencies was one of the real management factors that determine the occurrence or resolution of illegal land use.

### 5.3. Spatial-Temporal Distribution Characteristics of Illegal Land Use

According to the results of the previous research, China has always been promoting the construction of regional economies, such as the Yangtze River Economic Belt, the Central Plains Economic Zone, and the Pearl River Delta Economic Zone [39,40]. As the management and control of the unlimited expansion of construction land become increasingly stringent, the rapid economic-social development of these regions will inevitably lead to a series of illegal land use cases [41], the occurrence of illegal land use cases is more aggregate in line with theoretical logic. However, according to the calculation results of global Moran’s I, the global spatial autocorrelation of each variable was declining as a whole, and there was no spatial autocorrelation of each variable in 2017. The comparison between the characteristic variables of different illegal land use showed that VLDCY changed the slowest from agglomeration distribution to random distribution, but CasesSTY changed the fastest from agglomeration distribution to random distribution; this showed that the level of control and resolution of illegal cases in various places was constantly improving, which has promoted the decoupling of economic-social development from the occurrence or resolution of illegal land use cases.

Based on the calculation results of local Moran’s I, from the perspective of time, the L-L agglomeration units and the H-H agglomeration units were decreasing, this indicated that the probability of illegal land use cases was increasing in units where illegal land use cases had rarely occurred (L-L agglomeration). Moreover, this increase was different-in-different provinces. On the other hand, it showed that the level of the occurrence and resolution of regional illegal land use cases were constantly improving in the units of H-H agglomeration. From the perspective of spatial distribution, the L-L agglomeration units of illegal land use were mainly in the central-western regions, which was obviously related to the local economic-social development level. With the continuous improvement of the economic-social development level in the central-western regions, the incidence of illegal land use cases was constantly increasing, but the speed of resolution about illegal land use cases was constantly improving at the same time. The H-H agglomeration units of illegal land were mainly concentrated in the central-eastern regions, but there were fewer evaluation units involved as a whole, which showed that although the overall level of economic-social development in the central-eastern regions was relatively high, the ability to control and solve illegal land use increase more quickly than this.

### 5.4. Driving Mechanism of Structural Factors on the Characteristics of Illegal Land Use

From the factor detection results, CasesULY was closely related to Rp and Pnsch, which showed that the key to the settlement of illegal land use cases that were difficult to solve lied in the farmers’ cognition and the education level of the entire society. Therefore, this paper believes that effective civic education on the adverse effects of illegal land use can prevent illegal land use. VaTin, Up, TNreden, ALpurchTY, LTpTY and Tarede all had a greater impact on VLDCY, which showed that the tertiary industry, the level of urbanization and the continuous development of the land market drove the occurrence of illegal land use cases, resulting in a continuous increase in the number of illegal land use cases. The factors that drove CasesSTY were highly consistent with the impact on VLDCY, which showed that the overall resolution of illegal land use cases was consistent with the law of the discovery of illegal land use cases and that the overall level of illegal land management in China was relatively high. However, VaSin, VaTin, and Up had stronger explanatory power for CLICULY, which showed that the increase of secondary industry, tertiary industry and urban population objectively increased the difficulty of solving illegal land use cases involving cultivated land. Therefore, it was necessary to strengthen the illegal land use behaviors that may arise from the continuous development of these three aspects and control them in the embryonic stage in the actual management.

From the results of interaction detection, each driving factor had the strongest interaction effect on VLDCY and CasesSTY, and each driving factor had a relatively weak interaction effect on CasesULY and CLICULY. The results showed that the occurrence or resolution of illegal land use cases was affected by multiple factors, rather than a single factor, but there were also core interaction driving factors. Therefore, the direction and level of economic-social development will inevitably result in illegal land use cases, but in the actual management process, managers need to pay attention to the occurrence of illegal land use cases that may be caused by core interaction driving factors.

From the results of ecological exploration, the driving factors that affected the spatial distribution of the characteristic variables of illegal land use were basically the same as the results of factor detection; this showed that the level and structure of economic and social development and the level of the development of the land market not only affected the occurrence of illegal land use cases, the settlement of illegal land use cases, and the unresolvable damage caused by illegal land use cases to cultivated land at this stage, but also determined the location where the illegal land use case occurred or resolved.

## 6. Conclusions

The characteristics of illegal land use cases are multidimensional; in order to effectively control the serious impact of illegal land use, we must not only prevent the occurrence of illegal land use cases but also promote the settlement of illegal land use cases and reduce the area of cultivated land involved in illegal land use cases. Second, the management level of land resources management agencies in most regions was constantly improving, which promoted the settlement of illegal land use cases and prevented the occurrence of illegal land use cases. Third, a series of rules, regulations, policies and measures established by the central government and local governments at all levels in recent years to control the occurrence of illegal land use, promote the settlement of illegal land use and reduce the encroachment of illegal land had played a good role.

Different characteristics of illegal land use are affected by various driving factors in different degrees. The difficult-to-solve illegal land use cases were greatly affected by the number of the rural population and the education level of the whole society. The number of illegal land use cases where discovered, and the number of illegal land use cases were resolved in that year were greatly affected by the behavior of the tertiary industry and the land market. The difficult-to-solve illegal land use cases involving the area of cultivated land were greatly affected by the secondary industry, the tertiary industry and the urban population. Therefore, in the process of local socioeconomic development, we should always pay attention to the impact of local socioeconomic structure and land market behavior on illegal land use cases from a comprehensive perspective.

## Figures and Tables

**Table 1:** Characteristics variables of illegal land use in China.

Variables	Definition	Sources of Data
NumLRMA	Number of land resources management agencies	CLRSY/CLRA
CasesULY	Cases unsettled from last year	CLRSY/CLRA
LAICULY	Land area involved in cases unsettled from last year, unit: hm^2^	CLRSY/CLRA
CLICULY	Cultivated land involved in cases unsettled from last year, unit: hm^2^	CLRSY/CLRA
VLDCY	Violations of law discovered in the current year	CLRSY/CLRA
LAIVLDCY	Land area involved in violations of law discovered in the current year, unit: hm^2^	CLRSY/CLRA
CLIVLDCY	Cultivated land involved in violations of law discovered in the current year, unit: hm^2^	CLRSY/CLRA
CasesSTY	Cases settled this year	CLRSY/CLRA
LAICSTY	Land area involved in cases settled this year, unit: hm^2^	CLRSY/CLRA
CLICSTY	Cultivated land involved in cases settled this year, unit: hm^2^	CLRSY/CLRA

**Table 2:** Variables of the driving factors about illegal land use.

Classification	Variables	Definition	Sources of Data
Economic Structure	VaPin	Value-added of primary industry	CSY/SYDP/CRSY
	VaSin	Value-added of secondary industry	CSY/SYDP/CRSY
	VaTin	Value-added of tertiary industry	CSY/SYDP/CRSY
Social Structure	Up	Urban population	CSY/SYDP/CRSY
	Rp	Rural population	CSY/SYDP/CRSY
	Pnsch	Population of no schooling	CSY/SYDP/CRSY
	Upd	Urban population density	CSY/SYDP/CRSY
	Ur	Unemployment rate	CSY/SYDP/CRSY
Land market behavior	TNreden	Total number of real estate development enterprises (number)	CRESY/SYDP
	ALpurchTY	Area of land purchased this year (square meters)	CRESY/SYDP
	LTpTY	Land transaction price this year (ten thousand yuan)	CRESY/SYDP
	Tarede	Total assets of real estate development enterprises (ten thousand yuan)	CRESY/SYDP

**Table 3:** Interactive types of two controlled variables on dependent variables.

Decision Rules	Types of Interaction
*q*(X_1_ ∩ X_2_) < Min(*q*(X_1_), *q*(X_2_))	Nonlinear attenuation
Min(*q*(X_1_), *q*(X_2_)) < *q*(X_1_ ∩ X_2_) < Max(*q*(X_1_), *q*(X_2_))	Single-factor nonlinear attenuation
*q*(X_1_ ∩ X_2_) > Max(*q*(X_1_), *q*(X_2_))	Two-factor enhancement
*q*(X_1_ ∩ X_2_) = *q*(X_1_) + *q*(X_2_)	Independent
*q*(X_1_ ∩ X_2_) > *q*(X_1_) + *q*(X_2_)	Nonlinear enhancement

**Table 4:** Correlation coefficient matrix between characteristics variables of illegal land use.

Variables	NumLRMA	CasesULY	VLDCY	CasesSTY	LAIVLDCY	LAICSTY	LAICULY	CLIVLDCY	CLICSTY	CLICULY
NumLRMA	1.000									
CasesULY	0.195 ***	1.000								
	0.000									
VLDCY	0.262 ***	0.377 ***	1.000							
	0.000	0.000								
CasesSTY	0.258 ***	0.340 ***	0.861 ***	1.000						
	0.000	0.000	0.000							
LAIVLDCY	0.287 ***	0.133 ***	0.479 ***	0.533 ***	1.000					
	0.000	−0.006	0.000	0.000						
LAICSTY	0.274 ***	0.139 ***	0.451 ***	0.573 ***	0.952 ***	1.000				
	0.000	−0.004	0.000	0.000	0.000					
LAICULY	0.179 ***	0.306 ***	0.126 ***	0.140 ***	0.431 ***	0.322 ***	1.000			
	0.000	0.000	−0.009	−0.004	0.000	0.000				
CLIVLDCY	0.159 ***	0.149 ***	0.479 ***	0.574 ***	0.885 ***	0.894 ***	0.226 ***	1.000		
	0.000	−0.002	0.000	0.000	0.000	0.000	0.000			
CLICSTY	0.167 ***	0.159 ***	0.462 ***	0.608 ***	0.833 ***	0.902 ***	0.185 ***	0.964 ***	1.000	
	−0.001	−0.001	0.000	0.000	0.000	0.000	−0.001	0.000		
CLICULY	0.158 ***	0.365 ***	0.258 ***	0.295 ***	0.472 ***	0.414 ***	0.641 ***	0.490 ***	0.431 ***	1.000
	−0.001	0.000	0.000	0.000	0.000	0.000	0.000	0.000	0.000	

Notes: ***: *p* < 0.01.

**Table 5:** Global Moran’s I in China from 2004 to 2017.

Year	CasesULY		VLDCY		CasesSTY		CLICULY		NumLRMA	
	Moran’I	Z Value	Moran’I	Z Value	Moran’I	Z Value	Moran’I	Z Value	Moran’I	Z Value
2004	0.1367 *	1.7697	0.2736 *	2.8124	0.1663 *	1.8794	0.3035 *	3.1732	0.1428 *	1.5934
2005	0.2836 *	3.0867	0.2246 *	2.5266	0.1572 *	2.0548	0.1984 *	2.2223	0.1223 *	1.3936
2006	0.2859 *	2.9816	0.2046 *	2.2076	0.1762 *	2.0585	0.2961 *	3.1465	0.0083	0.4937
2007	0.3803 *	3.8534	0.1527 *	1.7461	0.1143 *	1.4837	0.2295 *	2.4237	0.0016	0.4063
2008	0.1676 *	1.8849	0.1963 *	2.1919	0.0539	1.0774	0.1867 *	2.1439	0.0055	0.4539
2009	0.0731	1.0037	0.2523 *	2.7682	0.2713	2.7771	0.1234 *	1.5021	−0.0232	0.0886
2010	0.0249	0.5618	0.2167 *	2.4173	0.1788	1.9852	0.1094 *	1.8741	−0.0144	0.1976
2011	0.0710	1.0124	0.2351 *	2.6159	−0.0108	0.2493	−0.0136	0.1706	−0.0215	0.0817
2012	−0.0443	−0.1398	0.1517 *	1.9124	0.1399	1.6022	0.1425	1.7692	−0.0071	0.2158
2013	−0.0041	0.2706	0.0594	0.8435	0.1908	2.0739	−0.0039	0.2761	0.0663	0.8852
2014	−0.0103	0.1969	0.0382	0.6339	0.0579	0.8332	0.0831	1.2939	0.0402	0.6467
2015	−0.0098	0.2455	0.1248	1.4808	0.1233	1.4388	0.0032	0.7124	0.0045	0.3213
2016	−0.0197	0.1664	0.0981	1.4554	0.0773	1.0217	−0.0535	−0.2761	−0.0141	0.1514
2017	−0.0064	0.4023	0.0158	0.6675	−0.0035	0.3576	−0.0576	−0.0576	−0.0056	0.2297

* represents the appearance of spatial agglomeration.

**Table 6:** Results of factor detection of driving factors on illegal land use in China.

Variables	CasesULY	VLDCY	CasesSTY	CLICULY
VaPin	0.3055	0.4170	0.4386	0.3933
VaSin	0.1641	0.4118	0.3867	0.6229
VaTin	0.1848	0.8797	0.8722	0.6302
Up	0.2665	0.8845	0.8412	0.6132
Rp	0.6965	0.4492	0.3476	0.3976
Pnsch	0.8107	0.3127	0.2735	0.5017
Upd	0.3548	0.2471	0.2718	0.2383
Ur	0.2691	0.3244	0.4206	0.3191
TNreden	0.3400	0.8768	0.8401	0.5134
ALpurchTY	0.2190	0.8959	0.8491	0.5270
LTpTY	0.2524	0.9139	0.8529	0.3926
Tarede	0.2332	0.8888	0.8601	0.3909

**Table 7:** Interactions between driving factors of CasesULY on illegal land use in China.

	Up	Rp	VaPin	VaSin	VaTin	Pnsch	Upd	TNreden	ALpurchTY	LTpTY	Tarede	Ur
Up	0.2665											
Rp	0.9181	0.6965										
VaPin	0.5669	0.9666	0.3055									
VaSin	0.3537	0.9354	0.5893	0.1641								
VaTin	0.3424	0.9171	0.5650	0.2853	0.1848							
Pnsch	0.9210	0.9136	0.9167	0.9653	0.9229	0.8107						
Upd	0.9979 *	0.9676	0.9527	0.9720	0.9681	0.9996 *	0.3548					
TNreden	0.9358	0.9338	0.9798	0.5915	0.9390	0.9387	0.9709	0.3400				
ALpurchTY	0.5100	0.9349	0.5790	0.5090	0.3529	0.9327	0.9934	0.9565	0.2190			
LTpTY	0.5028	0.9426	0.5448	0.5368	0.4985	0.9211	0.9668	0.9460	0.5041	0.2524		
Tarede	0.5449	0.9909	0.5772	0.5547	0.5440	0.9640	0.9597	0.5158	0.5640	0.5156	0.2332	
Ur	0.9970 *	0.9972 *	0.9429	0.6226	0.9967 *	0.9455	0.9981 *	0.4628	0.9978 *	0.9953	0.5648	0.2691

* represents the strongest interaction among all types.

**Table 8:** Interactions between driving factors of VLDCY on illegal land use in China.

	Up	Rp	VaPin	VaSin	VaTin	Pnsch	Upd	TNreden	ALpurchTY	LTpTY	Tarede	Ur
Up	0.8845											
Rp	0.9505	0.4492										
VaPin	0.9515	0.9479	0.4170									
VaSin	0.9499	0.9707	0.9953 *	0.4118								
VaTin	0.9280	0.9278	0.9269	0.9216	0.8797							
Pnsch	0.9520	0.9451	0.5172	0.9688	0.9415	0.3127						
Upd	0.9948 *	0.9352	0.9561	0.9256	0.9540	0.9769	0.2471					
TNreden	0.9216	0.9286	0.9653	0.9278	0.9092	0.9406	0.9784	0.8768				
ALpurchTY	0.9670	0.9577	0.9803	0.9581	0.9510	0.9607	0.9846	0.9481	0.8959			
LTpTY	0.9445	0.9914	0.9486	0.9958	0.9503	0.9591	0.9926	0.9420	0.9508	0.9139		
Tarede	0.9342	0.9862	0.9602	0.9272	0.9183	0.9583	0.9335	0.9336	0.9623	0.9399	0.8888	
Ur	0.9966 *	0.9800	0.5397	0.9762	0.9713	0.5546	0.9621	0.9695	0.9919 *	0.9953 *	0.9511	0.3244

* represents the strongest interaction among all types.

**Table 9:** Interactions between driving factors of CasesSTY on illegal land use in China.

	Up	Rp	VaPin	VaSin	VaTin	Pnsch	Upd	TNreden	ALpurchTY	LTpTY	Tarede	Ur
Up	0.8412											
Rp	0.9183	0.3476										
VaPin	0.9456	0.9189	0.4386									
VaSin	0.9565	0.9797	0.9898 *	0.3867								
VaTin	0.9205	0.9411	0.9532	0.9327	0.8722							
Pnsch	0.9174	0.8812	0.5184	0.9553	0.9647	0.2735						
Upd	0.9954 *	0.9631	0.9802	0.9421	0.9742	0.9862	0.2718					
TNreden	0.9018	0.9054	0.9316	0.9332	0.9146	0.9334	0.9795	0.8401				
ALpurchTY	0.9616	0.9625	0.9516	0.9356	0.9427	0.9378	0.9754	0.9642	0.8491			
LTpTY	0.9244	0.9408	0.9683	0.9644	0.9433	0.9209	0.9710	0.9369	0.9163	0.8529		
Tarede	0.9368	0.9580	0.9840	0.9455	0.9196	0.9294	0.9521	0.9292	0.9791	0.9324	0.8601	
Ur	0.9897 *	0.9745	0.5601	0.9659	0.9668	0.5767	0.9945	0.9631	0.9888 *	0.9850	0.9870	0.4206

* represents the strongest interaction among all types.

**Table 10:** Interactions between driving factors of CLICULY on illegal land use in China.

	Up	Rp	VaPin	VaSin	VaTin	Pnsch	Upd	TNreden	ALpurchTY	LTpTY	Tarede	Ur
Up	0.6132											
Rp	0.7997	0.3976										
VaPin	0.8731	0.8988	0.3933									
VaSin	0.7541	0.8503	0.9577	0.6229								
VaTin	0.6720	0.8093	0.8498	0.7348	0.6302							
Pnsch	0.8469	0.8471	0.7289	0.9545	0.8563	0.5017						
Upd	0.9938 *	0.9272	0.9131	0.9281	0.9086	0.9637	0.2383					
TNreden	0.7283	0.7797	0.9326	0.7685	0.7355	0.8334	0.8269	0.5134				
ALpurchTY	0.8531	0.8867	0.9162	0.8687	0.8329	0.8575	0.9052	0.8232	0.5270			
LTpTY	0.7817	0.9105	0.9260	0.9184	0.7953	0.7896	0.9785	0.8369	0.7594	0.3926		
Tarede	0.7690	0.9410	0.8632	0.8519	0.7375	0.8272	0.9356	0.7158	0.8629	0.8133	0.3909	
Ur	0.9719 *	0.9965 *	0.9451	0.9149	0.9667	0.9414	0.9480	0.8855	0.9952	0.7083	0.9001	0.3191

* represents the strongest interaction among all types.

**Table 11:** Results of the ecological detector between driving factors of CasesULY on illegal land use in China.

	Up	Rp	VaPin	VaSin	VaTin	Pnsch	Upd	TNreden	ALpurchTY	LTpTY	Tarede	Ur
Up												
Rp	Y											
VaPin	N	N										
VaSin	N	N	N									
VaTin	N	N	N	N								
Pnsch	Y	N	Y	Y	Y							
Upd	N	N	N	N	N	N						
TNreden	N	N	N	N	N	N	N					
ALpurchTY	N	N	N	N	N	N	N	N				
LTpTY	N	N	N	N	N	N	N	N	N			
Tarede	N	N	N	N	N	N	N	N	N	N		
Ur	N	N	N	N	N	N	N	N	N	N	N	

**Table 12:** Results of the ecological detector between driving factors of VLDCY.

	Up	Rp	VaPin	VaSin	VaTin	Pnsch	Upd	TNreden	ALpurchTY	LTpTY	Tarede	Ur
Up												
Rp	N											
VaPin	N	N										
VaSin	N	N	N									
VaTin	N	Y	Y	Y								
Pnsch	N	N	N	N	N							
Upd	N	N	N	N	N	N						
TNreden	N	Y	Y	Y	N	Y	Y					
ALpurchTY	N	Y	Y	Y	N	Y	Y	N				
LTpTY	N	Y	Y	Y	N	Y	Y	N	N			
Tarede	N	Y	Y	Y	N	Y	Y	N	N	N		
Ur	N	N	N	N	N	N	N	N	N	N	N	

**Table 13:** Results of the ecological detector between driving factors of CasesSTY.

	Up	Rp	VaPin	VaSin	VaTin	Pnsch	Upd	TNreden	ALpurchTY	LTpTY	Tarede	Ur
Up												
Rp	N											
VaPin	N	N										
VaSin	N	N	N									
VaTin	N	Y	Y	Y								
Pnsch	N	N	N	N	N							
Upd	N	N	N	N	N	N						
TNreden	N	Y	Y	Y	N	Y	Y					
ALpurchTY	N	Y	Y	Y	N	Y	Y	N				
LTpTY	N	Y	Y	Y	N	Y	Y	N	N			
Tarede	N	Y	Y	Y	N	Y	Y	N	N	N		
Ur	N	N	N	N	N	N	N	N	N	N	N	

**Table 14:** Results of the ecological detector between driving factors of CLICULY.

	Up	Rp	VaPin	VaSin	VaTin	Pnsch	Upd	TNreden	ALpurchTY	LTpTY	Tarede	Ur
Up												
Rp	N											
VaPin	N	N										
VaSin	N	N	Y									
VaTin	N	Y	Y	N								
Pnsch	N	N	N	N	N							
Upd	N	N	N	N	N	N						
TNreden	N	N	N	N	N	N	N					
ALpurchTY	N	N	N	N	N	N	Y	N				
LTpTY	N	N	N	N	N	N	N	N	N			
Tarede	N	N	N	N	N	N	N	N	N	N		
Ur	N	N	N	N	N	N	N	N	N	N	N	

## Data Availability

The raw data supporting the conclusions of this article will be made available by the authors: without undue reservation.
